# Low microbial abundance and community diversity in the egg capsule of the oviparous cloudy catshark (*Scyliorhinus torazame*) during oviposition

**DOI:** 10.1111/1758-2229.70025

**Published:** 2024-10-22

**Authors:** Wataru Takagi, Ayami Masuda, Koya Shimoyama, Kotaro Tokunaga, Susumu Hyodo, Yuki Sato‐Takabe

**Affiliations:** ^1^ Laboratory of Physiology, Atmosphere and Ocean Research Institute The University of Tokyo Kashiwa Chiba Japan; ^2^ Ibaraki Prefectural Oarai Aquarium Oarai Ibaraki Japan; ^3^ Marine Microbiology, Atmosphere and Ocean Research Institute The University of Tokyo Kashiwa Chiba Japan; ^4^ Department of Food and Nutrition Japan Women's University Bunkyo‐ku Tokyo Japan; ^5^ School of Economics Senshu University Kawasaki Kanagawa Japan

## Abstract

Vertebrate embryos are protected from bacterial infection by various maternally derived factors, yet little is known about the defence mechanisms in elasmobranchs. This study aimed to characterize the intracapsular environment of freshly laid eggs of the oviparous catshark (*Scyliorhinus torazame*) by investigating the microbial abundance and microbiota to understand its potential contribution to embryonic defence. The egg capsule of oviparous elasmobranchs is tightly sealed until pre‐hatching, after which seawater flows into the capsule, exposing the embryos to the surrounding seawater. We found that early embryos were highly vulnerable to environmental pathogens, suggesting that the embryos are somehow protected from infection before pre‐hatching. Indeed, the intracapsular environment of freshly laid eggs exhibited significantly low bacterial density, maintained until pre‐hatching. Furthermore, the microbiome inside eggs just after oviposition differed markedly from those of rearing seawater and adult oviducal gland epithelia; these eggs were predominantly populated by an unidentified genus of Sphingomonadaceae. Overall, this study provides compelling evidence that early embryos of oviparous cloudy catshark are incubated in a clean intracapsular environment that potentially plays a significant role in embryonic development in oviparous elasmobranchs.

## Graphical abstract

This research reveals that embryos of the oviparous cloudy catshark (*Scyliorhinus torazame*) acquire pathogen resistance by Stage 30. The intracapsular environment of freshly laid eggs exhibits low microbial abundance and diversity. This clean environment persists until the early opening of the capsule, suggesting its critical role in protecting immunologically immature embryos. Notably, an unidentified genus of Sphingomonadaceae dominates the microbial community in the freshly laid egg.

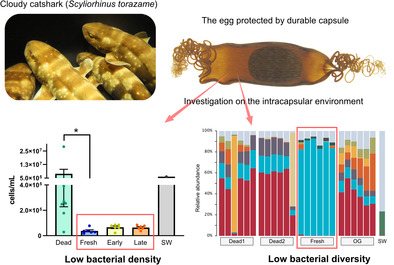

## INTRODUCTION

Elasmobranchs (sharks, skates and rays) exhibit remarkable diversity in reproductive strategies, ranging from oviparity to placental viviparity, accompanied by various modes of supplying embryonic nutrition (Luer & Wyffels, 2022; Wourms, 1977). Regardless of their reproductive mode, all elasmobranchs undergo internal fertilization via copulation, and their eggs are subsequently enclosed in a tertiary egg capsule, produced by the oviducal gland (also known as the egg‐shell gland or nidamental gland) (Hamlett et al., 1998). In most oviparous species, the incubation period spans at least several months, during which embryos develop outside the mother's body and rely solely on egg yolk for nutrition (Ballard et al., 1993; Musa et al., 2018; Wourms, 1977).

Oviparous elasmobranchs produce tough and durable egg capsules that protect the embryos against various challenges in the marine environment, such as mechanical stress and predation (Ballard et al., 1993; Kormanik, 1993; Musa et al., 2018). At the time of oviposition, both terminal ends of the capsule are tightly sealed by the solid egg jelly. The jelly gradually liquefies as development progresses (Ballard et al., 1993), and the capsule eventually opens during the mid‐period of development, an event known as ‘pre‐hatching’ (Ballard et al., 1993). The embryos can survive outside the capsule after the pre‐hatching, while the presence of a hole in the egg capsule wall of sufficient size to allow the influx of seawater will reportedly lead to the immediate death of the embryo before the pre‐hatching (Ballard et al., 1993). In such cases, infection with marine pathogenic bacteria is considered the primary cause of embryonic death. Indeed, this hypothesis is supported by reports that the addition of antibiotics to rearing buffered‐NaCl solution or seawater can extend the lifespan of early embryos outside the capsule in several oviparous shark species (Kuroda et al., 2021; Onimaru et al., 2018). However, when the embryos themselves acquire resistance to environmental pathogens and how they are immunologically protected before this acquisition remain largely unknown and has never experimentally tested.

The capsule wall is highly porous, with an estimated pore radius of 13.6 Å (= 1.36 nm) in *Scyliorhinus canicula* (Hornsey, 1978), which is significantly smaller than the average size of a bacterium (Nakai, 2020). The capsule wall of oviparous cartilaginous fishes is highly permeable to small molecules such as dissolved gases, water, urea, electrolytes and glucose (Hornsey, 1978; Kormanik, 1993; Takagi et al., 2017). In other words, the entry and exit of bacteria before the pre‐hatching period are likely minimal, whereas small molecules are frequently exchanged across the capsule wall. Furthermore, the egg capsule itself reportedly exhibits anti‐fouling properties (Thomason et al., 1996). These characteristics of the capsule wall are thus thought to prevent the invasion of pathogenic bacteria or fungi from the external environment.

In oviparous species, the inside of the capsule is filled with egg jelly surrounding the fertilized egg at oviposition. The jelly consists of three layers with distinct properties: a liquid layer closest to the embryo, a viscous colloid layer and a semi‐translucent solid layer (Wyffels et al., 2006). Unlike the function of the capsule wall, details regarding the function of egg jelly in this context remain unclear and debatable. Physical protection of the embryo is considered the primary role of egg jelly (Koob & Straus, 1998; Musa et al., 2018). Meanwhile, in other oviparous vertebrates, the substances surrounding the fertilized egg, such as hen's egg white, contain an abundance of bactericidal proteins that contribute to defence against pathogens (Giansanti et al., 2012). Antibacterial activity against several gram‐positive bacteria was also observed in an investigation of the egg jelly from *Scyliorhinus stellaris*, suggesting the presence of antimicrobial substances (Martinengo et al., 2024). However, these substance have not yet been identified in the jelly of any oviparous elasmobranchs (Lenain & Henderson, 2020).

Details regarding the bacterial flora, symbiotic interactions with the hosts, and types of pathogenic bacteria in elasmobranchs have recently begun to be reported (Perry et al., 2021). A pioneering study of the oviparous little skate, *Leucoraja erinacea*, characterized the microbiota within the egg capsule and demonstrated intergenerational vertical microbial transmission (Mika et al., 2021). However, while this previous work provided the first information regarding the microbiota of developing embryos in oviparous elasmobranch, the bacterial abundance within the capsule and whether and how the bacterial flora contributes to embryonic defence remain unclear.

Here, to further understand the defence strategies embryos adopt for their survival, we experimentally examined the pathogen resistance of developing embryos and investigated the intracapsular microbial environment in the eggs of the cloudy catshark, *Scyliorhinus torazame*. The bacterial density within the egg capsule was directly measured, and 16S rRNA metabarcoding analysis was also conducted to characterize the microbial community.

## EXPERIMENTAL PROCEDURES

### Animals

Adult female catsharks were transported from the Ibaraki Prefectural Oarai Aquarium to the Atmosphere and Ocean Research Institute and kept in 1000 and 3000 L holding tanks with recirculating natural seawater at 16°C, maintaining a constant photoperiod (12 h light:12 h dark). The egg‐laying cycle of catshark occurs approximately once every two or three weeks (Inoue et al., 2022). The fertilized eggs were manually collected from the cloaca of females, tagged to identify the date of oviposition, and subsequently placed in a separate tank away from adult individuals. Developmental stages were primarily identified according to the staging criteria of the closely related *S. canicula* (Ballard et al., 1993). The occurrence of pre‐hatching was visually determined by confirming whether air entered the capsules when taken out of water. Following the previous study (Honda et al., 2020), Stage 31, the pre‐hatching stage, was subdivided into stages 31E and 31 L based on whether the eggs were pre‐hatched. All animal experiments were approved by the Animal Ethics Committee of the Atmosphere and Ocean Research Institute of the University of Tokyo (P19‐2). The present study was carried out in compliance with the Animal Research: Reporting of In Vivo Experiments (ARRIVE) guidelines.

### Survival test

To test the resistance of embryos at different developmental stages to environmental pathogenic bacteria, survival test was conducted. The embryos just before pre‐hatching (st. 30–31E) and earlier embryos (st. 27–28) were carefully removed from the egg capsules and individually transferred to 60 mL rearing containers, each filled with seawater under the following conditions. The seawater originated from the coastal region of Oarai town, Ibaraki Prefecture, which is the habitat of catshark. One was supplemented with an antibiotic‐antifungal mixture (100× concentrated, penicillin 10,000 U/mL, streptomycin 10,000 μg/mL, amphotericin B 25 μg/mL, 0.85% saline, Nacalai Tesque, Kyoto, Japan) to achieve a final concentration of 0.2% (st. 31E, *N* = 6; st. 27–28, *N* = 6), while the other was without antibiotics (st. 31E, *N* = 5; st. 27–28, *N* = 5). The containers were maintained at 16°C in an air incubator. The culturing seawater was changed multiple times a week according to the previous study (Kuroda et al., 2021), and survival days were recorded. Statistical analysis was conducted using the survival package (Therneau & Grambsch, 2000) in R Studio (v. 2023.09.1.494) (Posit team, 2023; R Core Team, 2023).

### Bacterial abundance

**FIGURE 1:**
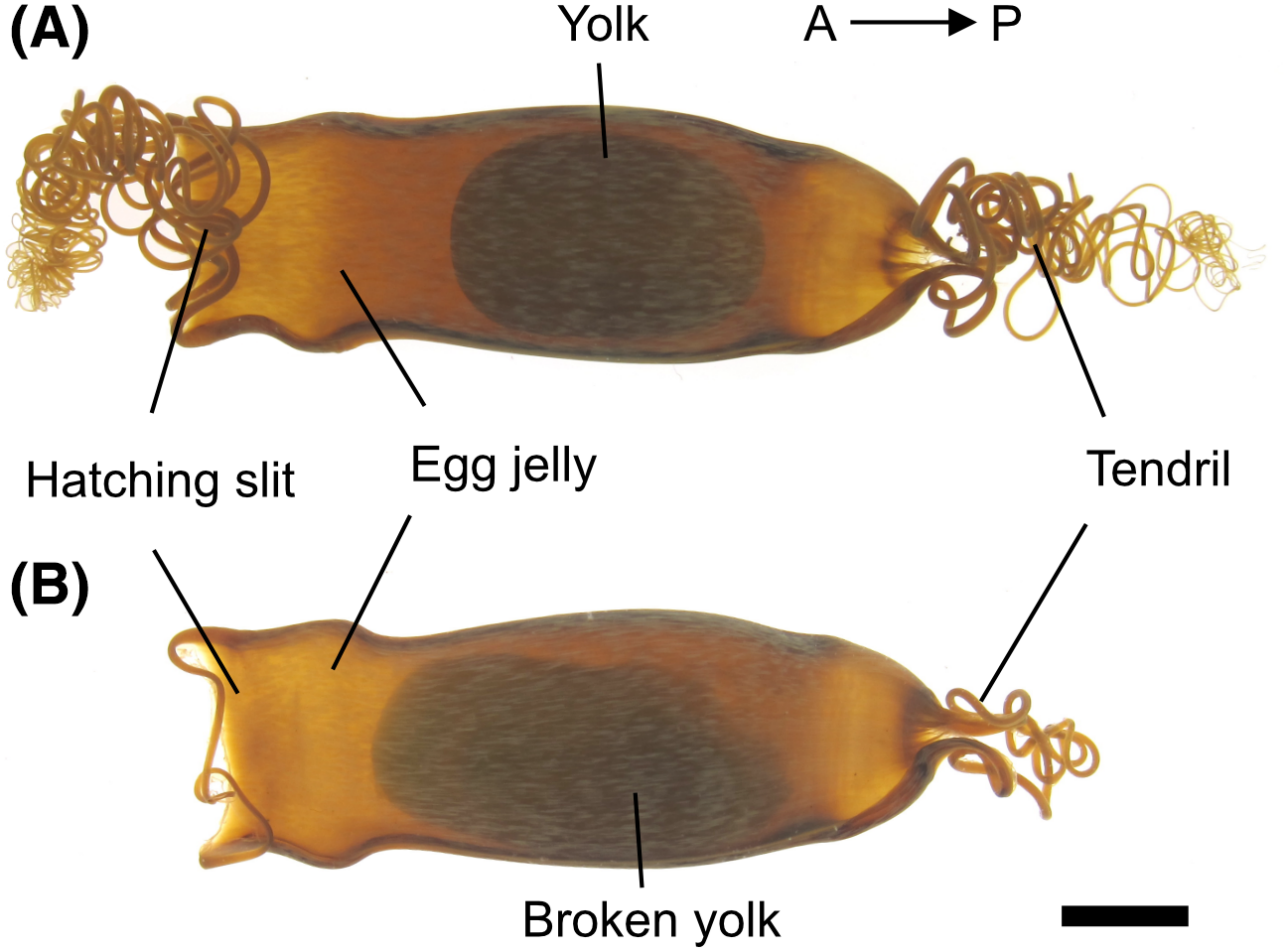
Eggs of cloudy catshark. The fertilized egg is encapsulated in the tough capsule filled with egg jelly. Arrows indicates the anterior–posterior axis with respect to the adult female. Prior to pre‐hatching period (before Stage 31E), the jelly plugs both the anterior and posterior ends of the capsule, preventing the entry of surrounding seawater. At pre‐hatching, the anterior end of the capsule (hatching slit) opens. (A) Freshly laid egg. (B) ‘Dead’ egg, containing yolk of which acellular vitelline membrane was broken. Scale bar, 1 cm.

The surfaces of all the eggs and dissecting instruments were sterilized just before sampling using paper towels soaked in 70% ethanol. The anterior end of the egg capsules was trimmed with sterilized scissors, and the egg jelly within the capsule was aseptically collected using disposable pipettes. Embryos in normally developing eggs become observable 1 month after oviposition when exposed to intense light from the outside. Meanwhile, in unfertilized or developmentally arrested eggs, for some reason, the egg yolk swells and leading to irregular disintegration (Figure 1B). In this study, such eggs that did not progress in development even after a month were designated as ‘dead eggs’. Total contents inside the egg capsule containing egg jelly and yolk were collected from the dead eggs (*N* = 7), while only aqueous egg jelly surrounding the embryos was collected from the eggs containing embryo (st. 27–28, *N* = 6; st. 30–31E, *N* = 6). For the freshly laid eggs, only viscous jelly was collected immediately after oviposition and homogenized in a sterilized stomacher bag (Sansei medical Co. Ltd., Kyoto, Japan) using a rubber roller (*N* = 6). Each sample whose density is assumed to be close to 1 kg/m^3^, was weighed and diluted with sterile distilled water to achieve a total volume of 1 mL. Undiluted 8 mL of seawater was also collected from the tank rearing all eggs examined in this study (*N* = 1). All samples were immediately fixed by adding 10% neutral buffered formalin (Fujifilm Wako Pure Chemical Corporation, Osaka, Japan) to achieve a final concentration of 1% in the dark at 4°C. Fixed samples were stained with 4′,6‐diamidino‐2‐phenylindole (DAPI) for 10 min in the dark and then filtered through Nuclepore black polycarbonate membrane filters (0.22 μm pore size, Merck Millipore, Darmstadt, Germany) with negative pressure (≤20 kPa). The filtered membranes were mounted on glass slide, covered with a coverslip using immersion oil (IMMOIL‐F30CC, Olympus, Tokyo, Japan), and gently pressed by thumbs to be flattened for observation. Under a fluorescent microscope, 20 random fields were selected from the entire membrane. In each field, the bacterial count within a defined area (2000 μm^2^ for samples with high bacterial abundance and 10,000 μm^2^ for other samples) was measured, and the total bacterial count on the membrane was calculated. Eventually, considering the volume of the original sample and dilution factor, the bacterial density per unit volume (cells/mL) was determined. Differences in the density between groups were statistically analysed by the Kruskal–Wallis test with GraphPad Prizm version 9.4.0 (San Diego, CA, USA).

### DNA extraction and 16S rRNA amplicon sequencing

To analyse the types of bacteria and their relative abundances in the samples, DNA was extracted from each sample and 16S rRNA amplicon sequencing was performed. Using the same procedure as mentioned above, egg jelly was collected from freshly laid eggs (*N* = 10) and dead eggs (1 month after oviposition, *N* = 6; 2.5 months after oviposition, *N* = 6). The timing 2.5 months after oviposition was chosen because many of the dead eggs would swell and explode if left longer than this. The sample of the dead eggs included egg yolk. Seawater (200 mL) from the tank rearing all eggs examined in this study was also collected and filtered through a 0.22 μm filter (*N* = 1). The female individuals (*N* = 6) were anaesthetised with 0.02% (w/v) ethyl 3‐aminobenzoate methanesulfonate (Sigma‐Aldrich, St. Louis, MO, USA), euthanized by decapitation and dissected. The oviducal gland was carefully incised using sterile scissors. Nuclease‐free water (Qiagen, Hilden, Germany) was then applied onto the inner epithelia of the gland, pipetted several times, after which the liquid was collected into microtubes. All samples were immediately snap‐frozen in liquid nitrogen and stored at −30°C until use.

Total DNA was extracted using the DNeasy Blood & Tissue Kit (Qiagen) following the manufacturer's instructions. The extracted DNA was subsequently sent to Genome–Lead Co., Ltd. (Kagawa, Japan) for library preparation, quality control and sequencing, according to Illumina's official protocol (Illumina, 2013). A first PCR reaction was performed with KAPA HiFi 2X Mastermix (KAPA) to amplify the V3–V4 region of 16S rRNA, using the following primer sets: forward primer: 5′‐TCGTCGGCAGCGTCAGATGTGTATAAGAGACAGCCTACGGGNGGCWGCAG‐3′, reverse primer: 5′‐GTCTCGTGGGCTCGGAGATGTGTATAAGAGACAGGACTACHVGGGTATCTAATCC‐3′. The PCR procedure was as follows: initial denaturation at 95°C for 3 min, 28–32 cycles of 95°C for 30 s, 55°C for 30 s and 72°C for 30 s in a reaction volume 25 μL. The number of PCR cycles was determined based on the amplification efficiency. The PCR products were purified and barcoded with index sequences by a second PCR. The concentrations of the purified PCR products were normalized and 301 bp × 2 paired‐end reads were sequenced on the Illumina MiSeq platform. The acquired raw data were deposited in the NCBI Sequence Read Archive (Accession No: DRR523913‐523937).

Community analysis of the reads was performed using the QIIME2 program (ver. 2023.9) (Bolyen et al., 2019) as described below. First, primer sequences were removed using the Cutadapt plug‐in (Martin, 2011), and the sequence reads potentially including erroneous bases were removed and clustered based on amplicon sequence variants (ASVs) at single‐nucleotide resolution using the DADA2 plug‐in (Callahan et al., 2016). The ASVs were taxonomically classified with an amplicon region (the V3‐V4 region of 16S rRNA)‐specific naïve Bayes classifier derived from 99% OTUs in SILVA rRNA database ver.138.1 using RESCRIPt (Quast et al., 2013; Robeson 2nd et al., 2021). The data were subsequently rarefied to the minimum number of reads (17,024 reads) to normalize the read counts across samples. The relative composition of bacterial groups was visualized at the genus level using taxonomy bar plots generated by QIIME2 program (Bolyen et al., 2019). Furthermore, Maaslin2 was used in R Studio to identify significantly different taxa across samples using unrarefied raw count data (Mallick et al., 2021; Posit team, 2023; R Core Team, 2023). The seawater sample was excluded for this analysis. For the multiple comparisons, CLR normalization, LOG transformation and a linear model (LM) were applied with a *q*‐value threshold of 0.05.

The alpha‐diversity of the bacterial community among the samples was determined by Pielou's eveness and Shannon indices, and differences in the diversity between groups except for the seawater sample were statistically analysed by the Kruskal–Wallis test (*q* < 0.01) using QIIME2. The beta‐diversity of the bacterial community among the groups was determined by the Bray–Curtis index, and the significant differences between groups were analysed by the PERMANOVA test (*q* < 0.01) using QIIME2. All the statistical analyses were followed by Benjamini–Hochberg false discovery rate correction. Both QIIME2 and R Studio with the ggplot2 package were used for data visualization (Bolyen et al., 2019; Posit team, 2023; R Core Team, 2023).

## RESULTS

### Embryo survival rate in the extracapsular environment

**FIGURE 2:**
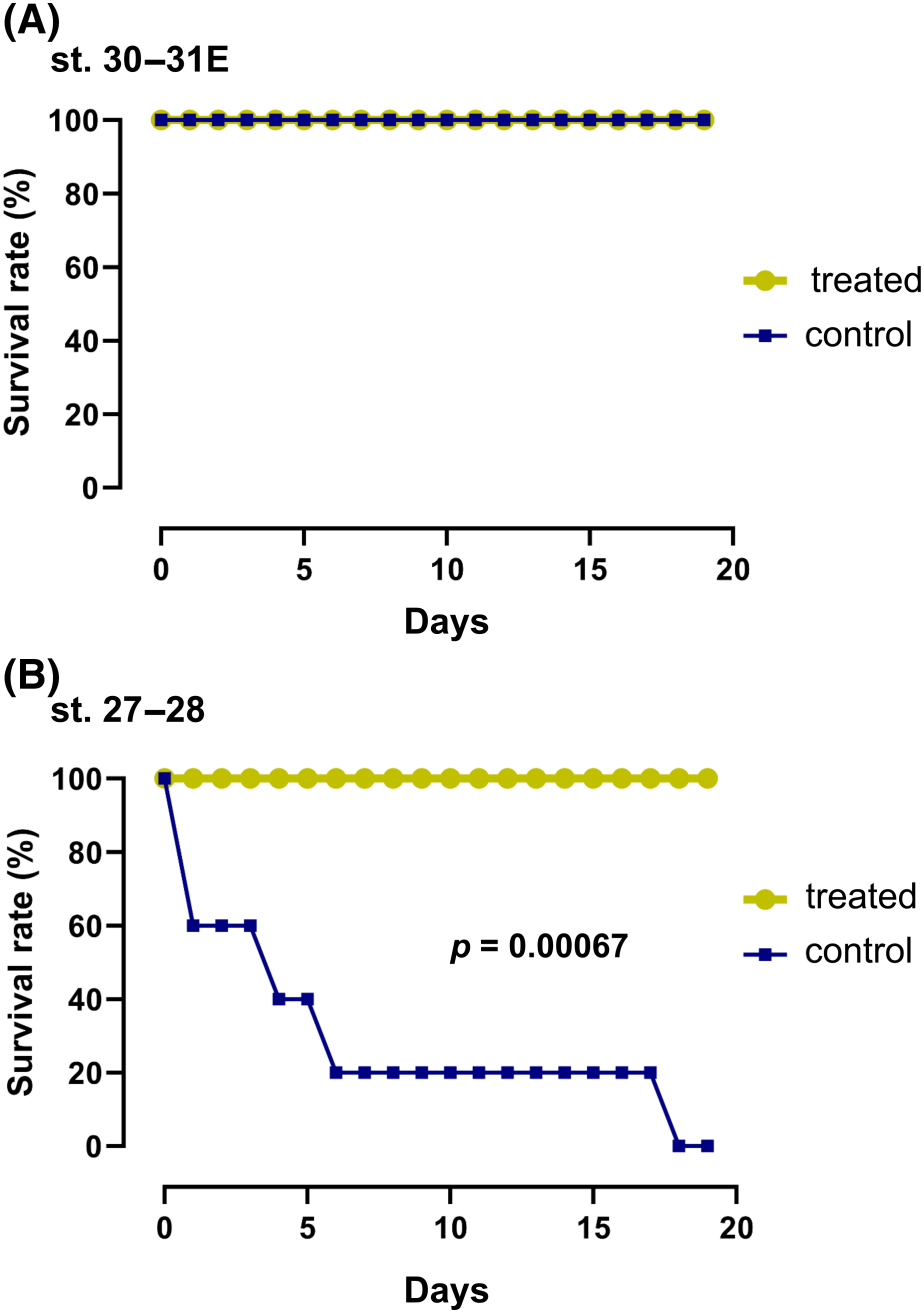
Embryo survival rate of catshark outside the capsule at two different developmental stages. The embryos were reared in natural seawater with (treated) or without (control) antibiotics. (A) Embryos at later stage (st. 30–31E). (B) Embryos at earlier stage (st. 27–28). At earlier stage, a significant difference was observed between the treated and control groups (*p* = 0.00067).

Developing catshark embryos were removed from the capsule and reared in a separate container filled with natural seawater with or without antibiotics (Figure 2). Embryos just prior to the pre‐hatching period (st. 30–31E) survived for at least 20 days in seawater outside the capsule, regardless of the presence of antibiotics (Figure 2A). Earlier embryos (st. 27–28) survived for 20 days in seawater supplemented with antibiotics, whereas the survival rate dropped to <50% within 4 days in the absence of antibiotics, and all embryos died within 18 days under this condition (Figure 2B). A statistical significant difference (*p* = 0.00067) was found in time until death between the antibiotic‐treated and untreated groups in early embryos.

### Abundance of microorganisms within the egg capsule

**FIGURE 3:**
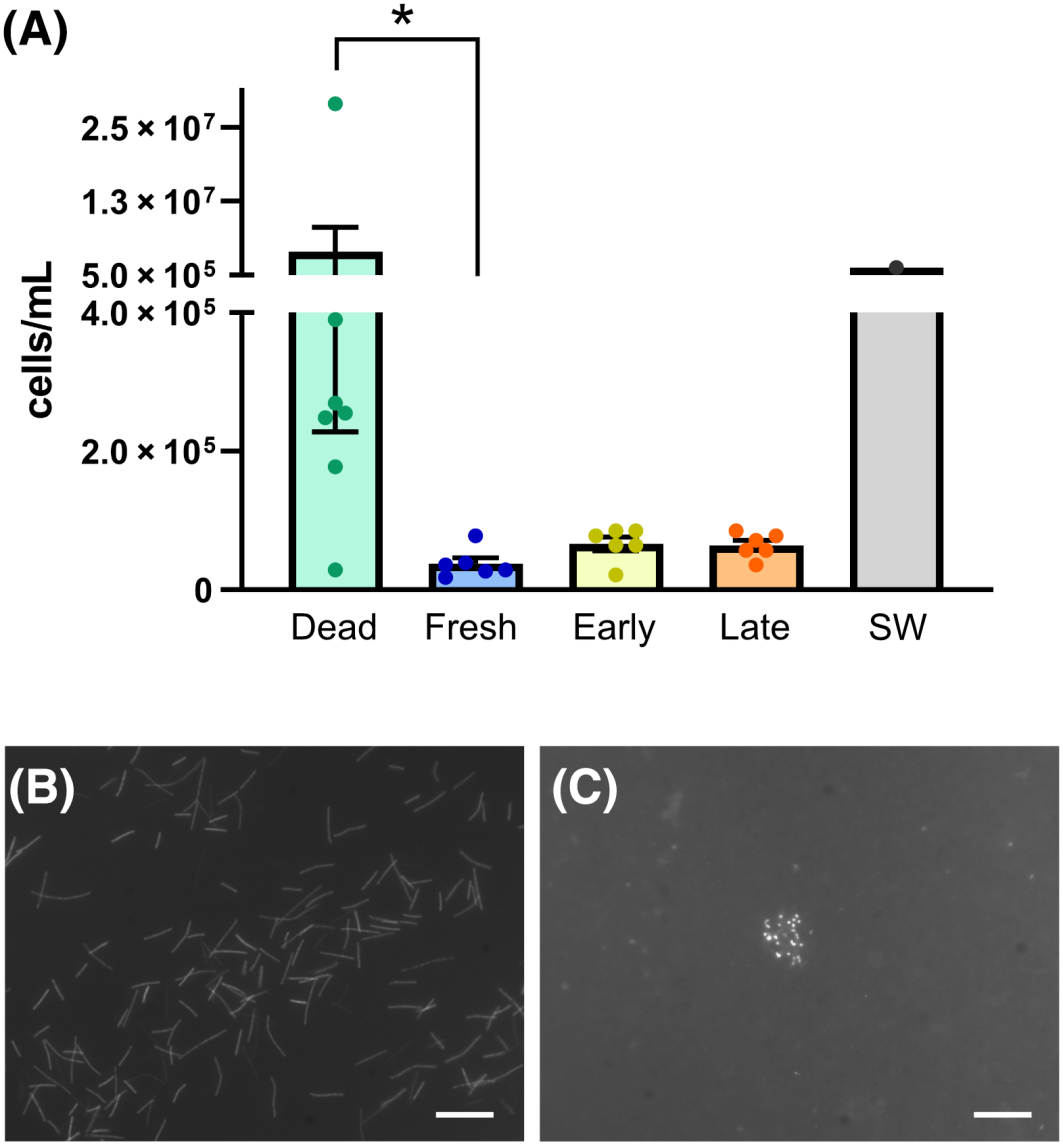
Microbial abundance inside the egg capsule of catshark. (A) Estimated numbers of bacteria per unit volume (cells/mL) in the contents inside the capsule. The egg conditions were as follows: dead (dead eggs, *N* = 7), fresh (freshly laid eggs, *N* = 6), early (eggs carrying embryos at stage 27–28, *N* = 6) and late (eggs carrying embryos at stage 30–31E, *N* = 6). The values are presented as the means ± SEM. The microbial density of the rearing seawater (SW) was also examined for comparison (*N* = 1). Asterisk indicates the statistical significance (*p* < 0.05). (B) Conspicuous bacterial outgrowth in selected dead eggs. (C) A representative bacterial aggregate found in freshly laid eggs. Scale bars, 10 μm.

The bacterial density in the internal environment of freshly laid eggs, dead eggs, eggs carrying embryos at two different stages and seawater was measured by counting the number of DAPI‐positive cells (Figure 3A). There were no statistically significant differences between the three groups: freshly laid eggs, eggs carrying embryos at st. 27–28 (Early), and eggs carrying embryos at st. 30–31E (Late). The density in the jelly of freshly laid eggs was >40 times lower (3.75 × 10^4^ ± 0.86 × 10^4^ cells/mL) than that of seawater in the container rearing the eggs (1.62 × 10^6^ cells/mL). Similarly, low values were obtained for the eggs carrying embryos, although these values were slightly greater than that of freshly laid eggs: st. 27–28, 6.61 × 10^4^ ± 0.98 × 10^4^ cells/mL; st. 30–31E and 6.38 × 10^4^ ± 0.73 × 10^4^ cells/mL.

In contrast, the highest average microbial density was observed in the intracapsular contents of dead eggs (4.25 × 10^6^ ± 4.02 × 10^6^ cells/mL, similar to that of seawater) (Figure 3A). The bacterial density in dead eggs varied markedly; one dead egg had an extremely low bacterial density, similar to that of freshly laid eggs (2.84 × 10^4^ cells/mL), whereas another dead egg had an exceptionally high density (2.83 × 10^7^ cells/mL), 17 times greater than that of seawater. In the latter case, a large number of rod‐shaped bacteria were observed in every observation field (Figure 3B). Interestingly, when the entire filtered membrane was thoroughly observed, we found bacterial aggregates in all samples of freshly laid eggs and eggs carrying embryos, but not in dead eggs or seawater (Figure 3C). The bacteria within the aggregates were not counted.

### Bacterial community within the eggs and oviducal gland epithelia

**FIGURE 4:**
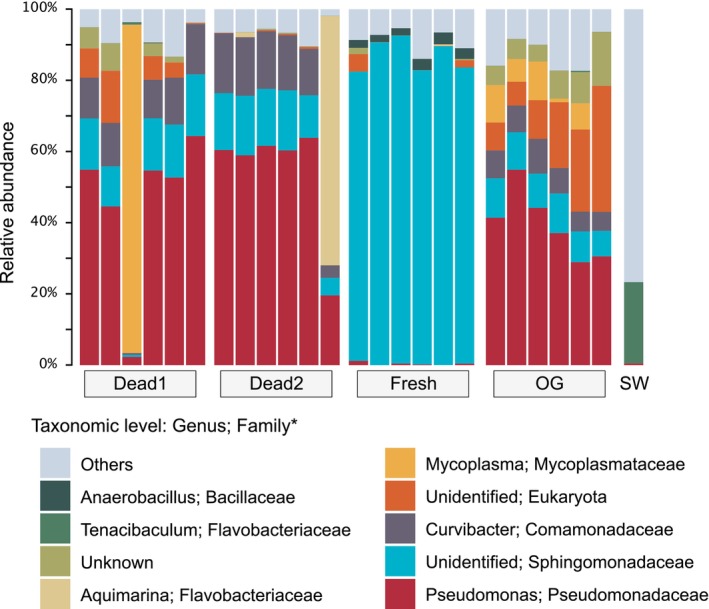
Taxonomic compositions at the genus level. Relative abundance of top 10 ASVs are shown in bar plots. Dead1, dead eggs 1 month after oviposition; dead2, dead eggs 2.5 months after oviposition; fresh, freshly laid eggs; OG, epithelia of oviducal gland; SW, seawater. *Note that ‘Eukaryota’ represents the domain level, not the family level.

All samples of DNA extracted from dead eggs, the oviducal gland epithelia, and seawater were successfully amplified and sequenced. However, 4 of 10 samples of freshly laid eggs were excluded from the examination due to insufficient PCR amplification for subsequent analyses. The ASV data were rarefied based on the minimum number of reads (17,024) and taxonomically classified into 3612 features (Figure S1). The assigned features were collapsed to taxonomic level 6, corresponding to ‘genus’ level, and the relative abundance of the top nine taxa was visualized (Figure 4).

In freshly laid eggs, an unidentified genus of the Sphingomonadaceae family (Pseudomonadota phylum, alpha‐Proteobacteria class) dominated the microbial community, comprising more than 81.8% in all six examined samples, with the highest sample reaching 92.1% (Figure 4). Although the unidentified Sphingomonadaceae genus was also present in all the other samples, except for seawater, the occupancy was less than 17.4%. The dominance of this genus in the freshly laid egg samples was significantly higher compared to the other samples (Figure S2). Meanwhile, 10 of 12 samples of dead eggs were dominated by *Pseudomonas* (57.6% on average, Proteobacteria phylum, gamma‐Proteobacteria class, Pseudomonadaceae family), followed by *Curvibacter* (13.8% on average, Pseudomonadota phylum, gamma‐Proteobacteria class, Comamonadaceae family). Furthermore, the bacterial communities in two individual samples of dead eggs were unique and distinct from those in other samples. The community in one dead eggs 1 month after oviposition exhibited a high occupancy of *Mycoplasma* (92.1%), whereas apredominance of *Aquimarina* (70.4%) was found in another dead egg 2.5 months after oviposition.

Similar to the microbiota of dead eggs 1 month after oviposition, the microbiota of the oviducal gland epithelia was dominated by *Pseudomonas*, *Curvibacter* and unidentified genera of the Sphingomonadaceae family. Although Pseudomonas was detected in all catshark‐derived samples, it was significantly lower in the freshly laid eggs (Figure S2). Eukaryote ASVs were identified in all oviducal gland samples, ranging from 6.5% to 35.4%, with an average of 17.1%. Contamination by eukaryotic nucleotides (8.5% on average) was also observed in four of six dead eggs 1 month after oviposition, but this contamination was <0.6% in dead eggs 2.5 months after oviposition. These eukaryotic ASVs in catshark‐derived samples were top‐hit‐matched to the *S. canicula* genomic sequence based on a BLASTn search, confirming host origin. In contrast to the communities in the catshark‐derived samples, the microbial community in the seawater sample was highly diverse and primarily dominated by *Tenacibaculum* (22.8%, Bacteroidota phylum, Bacteroidia class, Flavobacteriaceae family), followed by various taxa at <10% abundance.

### Bacterial diversity

**FIGURE 5:**
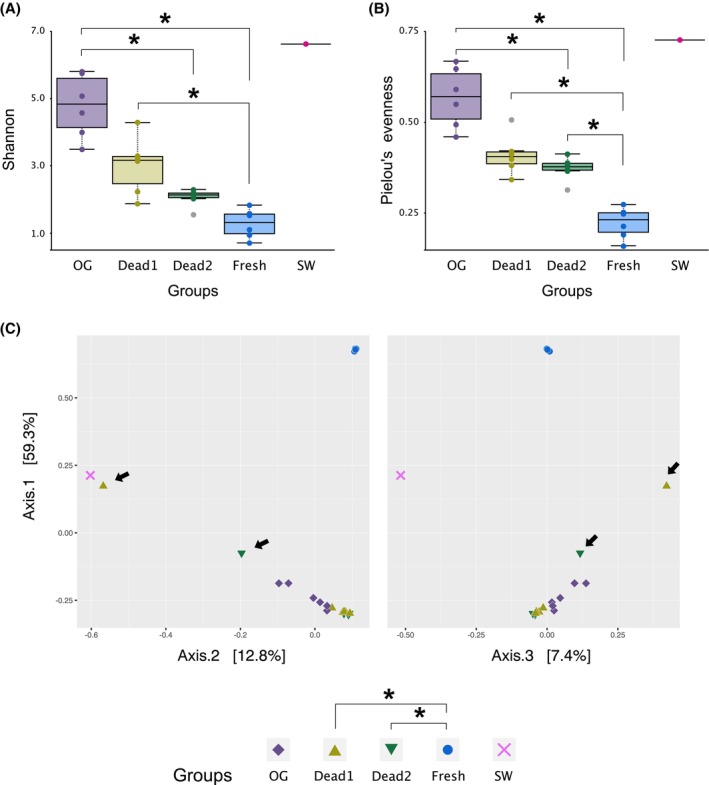
Results of alpha‐ and beta‐diversity. Asterisks indicate the statistical significance (*q* < 0.01). The abbreviation is the same as those in Figure 4. (A) Shannon and (B) Pielou's evenness were used for calculation of alpha‐diversity. Outlier samples are indicated by grey circles. (C) The Bray–Curtis dissimilarity index was used for beta‐diversity analysis. The PCoA plot shows the relatedness of microbial community among the examined groups. The *p*‐value from PERMANOVA test is 0.001. Arrows indicate two dead egg samples dominated by *Mycoplasma* and *Aquimarina*, respectively.

When analysing alpha‐diversity using both the Shannon and Pielou indices, freshly laid eggs showed the least diversity, whereas seawater exhibited the greatest diversity (Figure 5A,B). According to both the Shannon and Pielou indices, the alpha‐diversity of freshly laid eggs differed significantly from that of the oviducal gland epithelia and dead eggs, consistent with the results of the community analyses (Figures 4 and 5A,B). The oviducal gland epithelia exhibited the greatest diversity among catshark‐derived samples and differed significantly from that of dead eggs 2.5 months after oviposition. However, there was no differences in the diversity between the oviducal gland epithelia and dead eggs 1 month after oviposition. No significant differences were detected between the two groups of dead eggs (i.e., eggs of different time points after oviposition).

Taxonomic beta‐diversity was calculated using Bray–Curtis dissimilarity analysis, and the results are represented on principal coordinate axes (Figure 5C). All samples of freshly laid eggs clustered closely and showed low similarity with the other groups. Consistent with this result, statistical analysis revealed significant differences between freshly laid eggs and dead eggs. However, no significant difference in taxonomic diversity was observed between the oviducal gland epithelia and other samples. The two samples of dead eggs that exhibited unique bacterial compositions in the previous community analysis showed variability in spatial position (Figure 4, arrows in Figure 5C).

## DISCUSSION

### Embryonic immune functions in catshark

The inability of early embryos of oviparous elasmobranchs to survive outside the egg capsule has long been known (Ballard et al., 1993), and the addition of antibiotics to the rearing seawater is a well‐established empirical viability‐extending treatment (Kuroda et al., 2021; Onimaru et al., 2018). However, the point at which embryonic defence against pathogens is acquired during development has not been experimentally verified. In cloudy catshark, differences in the ability of early embryos to survive outside the egg capsule were observed between two developmental stages (st.27–28 and st.30–31E). Specifically, early embryos at st.27–28 could not survive outside the capsule without antibiotics, whereas embryos at st.30–31E were able to survive without antibiotics. This discrepancy is likely attributed to differences in embryonic resistance to potential pathogens, as all examined embryos survived with antibiotic treatment. Previous studies examining *S. torazame* demonstrated that the pre‐hatching period (st.31) is a critical time during which embryos develop respiratory, osmoregulatory and nutritional absorption functions (Honda et al., 2020; Takagi et al., 2017; Tomita et al., 2014). It is thus reasonable to consider that the period around developmental Stage 31 is also a key time for the acquisition of immune functions.

The transfer of passive immunity from mothers to embryos, in which maternal antibodies accumulated in egg yolk contribute to embryonic defence, has been documented in a variety of vertebrates (Hamal et al., 2006; Olsen & Press, 1997). In elasmobranchs, the presence of maternally derived immunoglobulins (7S IgM and IgNAR) in the egg yolk of viviparous nurse sharks (*Ginglymostoma cirratum*) suggests these immunoglobulins contribute to embryonic immunity (Haines et al., 2005). However, the inability of young catshark embryos at stages 27–28 to survive outside the capsule without antibiotics suggests that the embryos at this developmental stage likely lack the host immune defence to resist pathogenic bacteria present in seawater. It is unknown whether the oviparous catshark embryos possess maternal antibodies in their yolk as in viviparous nurse shark, but even if they do, the contribution to the embryonic defence appears to be minimal. Our data support the conclusion that early‐stage embryos of oviparous elasmobranchs lack resistance to pathogenic bacteria.

### Low microbial density within the egg capsule and its maintenance

Mika et al. (2021) characterized microbial communities in embryonic tissues, egg capsules and jelly in the oviparous little skate and demonstrated the vertical transfer of the microbiome from mother to embryo (Mika et al., 2021). However, the absolute bacterial abundance within the capsule of oviparous elasmobranchs has not been reported. Considering the results of the survival test, it can be assumed that a clean intracapsular environment, the presence of certain bacteriostatic substances within the jelly, or both, are important for survival of immunologically immature early embryos. Indeed, the jelly inside freshly laid eggs of catshark exhibited low bacterial density, which was maintained until pre‐hatching, and the bacterial community was unique compared with that of other samples, with low species richness. The intracapsular fluid of little skate at stage 0 (equivalent to the freshly laid egg in this study) and stage 16 showed very low species richness compared with later stages (Mika et al., 2021), in good accordance with our findings. Thus, a clean intracapsular environment for eggs at oviposition might be shared among oviparous elasmobranchs.

A tertiary egg coat that functions as a primary barrier for preventing the invasion of bacteria from the external environment is common among oviparous vertebrates. In chicken and Chinese soft‐shell turtle, the pore sizes of the membrane lining the eggshell is reportedly 25.2 and 20 nm, respectively (Kutchai & Steen, 1971; Yoshizaki et al., 2004). The chorion in teleost fishes also exhibits a submicron pore size, which is smaller than typical micron‐scale bacteria (Olivar, 1987). Likewise, the calculated pore size of the egg capsule wall in *S. canicula*, a close relative of *S. torazame*, is several nanometers (Hornsey, 1978). Considering these data together with our finding that the microbial compositions of both freshly laid eggs and dead eggs were distinct from that of seawater confirms that the catshark egg capsule wall functions as a structural barrier against the entry of microorganisms and thus likely contributes to the maintenance of a closed environment before pre‐hatching.

### Mechanism leading to generation of the unique intracapsular environment

Determining the mechanism by which the clean intracapsular environment is generated remains challenging. The aseptic condition within freshly laid eggs is a well‐known characteristic of avian species. Various physicochemical properties (e.g., temperature, pH and high viscosity) and an abundance of antimicrobial proteins (such as ovo‐transferrin and lysozyme) in the egg white of chicken inhibit bacterial growth (Baron et al., 2016; Giansanti et al., 2012). The presence of lysozyme in egg white has also been reported in reptiles (Gayen et al., 1977). In contrast, the egg jelly of oviparous elasmobranchs is a mucin hydrogel containing a substantial amount of water with a very low protein level (<0.001%) (Lenain & Henderson, 2020). Lenain and Henderson (2020) further demonstrated that the jelly does not affect the growth of bacteria isolated from the outer surface of the egg capsule, negating the contribution of the egg jelly to embryonic defence (Lenain & Henderson, 2020). However, it is not necessary that the jelly, which has no contact with bacteria outside the capsule, exhibit strong antibacterial activity. Indeed, the egg jelly from *Scyliorhinus stellaris* shows a slight inhibitory effect against gram‐positive bacteria (Martinengo et al., 2024). Thus, the jelly appears to play a bacteriostatic role in early development, given that the initial lowered bacterial abundance at the oviposition was maintained until pre‐hatching.

Within freshly laid eggs and eggs carrying developing embryos at two different stages, we observed bacterial aggregates, albeit infrequently, with the numbers remaining relatively stable throughout the examined developmental periods. The formation of bacterial aggregates has generally been reported as a response to infection, and a variety of factors mediate the induction of aggregation (Cai, 2020; Secor et al., 2018). In humans, for example, interactions between IgA secreted in saliva and bacterial surface components induce aggregation to promote clearance by blocking bacterial adhesion to oral tissue surfaces (Yamaguchi, 2004). Similarly, mucus secreted by the colon induces the aggregation of intestinal bacteria, preventing inflammation (Bergström et al., 2016). In addition, environments rich in polymers such as mucins and glycosaminoglycans also drive bacterial aggregation via entropic forces (Secor et al., 2018; Secor et al., 2022). Therefore, the formation of aggregates found inside the capsule might be induced by the mucin hydrogel and its degradation products, thereby suppressing bacterial activity and/or proliferation before pre‐hatching. Further research is warranted to explore the potential bacteriostatic action of the substance within the jelly.

A potential contributor to reduction in intracapsular bacterial biomass before oviposition is the oviducal gland, the site of both fertilization and encapsulation in elasmobranchs (Hamlett et al., 1998; Wourms, 1977). Production of bactericidal or bacteriostatic substances in adult reproductive organs is well documented among vertebrates (Iida et al., 2021; King et al., 2003; Yoshimura & Barua, 2017). Recent transcriptomic and proteomic analyses of the uterine fluid of viviparous red stingray (*Hemitrygon akajei*) revealed the presence of antimicrobial transferrins and dermicidin throughout the examined gestational periods (Kina et al., 2021). It is thus plausible that bactericidal substances are secreted from the oviducal gland of catshark to sterilize the luminal epithelium, thereby minimizing pathogen contamination of the capsule during the process of encapsulation.

It should be noted that the adult female animals dissected in this study were not in the state of forming egg capsules due to difficulties in identifying the timing of fertilization and subsequent egg encapsulation. The microbiome of oviducal gland epithelia during the encapsulation process may thus be different from what we observed and possibly similar to that of freshly laid eggs. The use of plasma progesterone concentration as an indicator of ovulation might allow us to obtain females just at the time of egg encapsulation (Inoue et al., 2022; Shimoyama et al., 2023). Examining the changes in microbial community of the oviducal gland epithelia throughout the reproductive cycle would help explain the differences in microbiome between the freshly laid eggs and oviducal gland epithelia and provide insights into the mechanism by which the abundance and diversity of bacteria inside the capsule are reduced before oviposition.

### Relationship between the microbiota and embryonic growth

As in other vertebrates, interactions between the host and microbes have been predicted in elasmobranchs (Perry et al., 2021). However, further research is still needed to understand what constitutes the core microbiota of elasmobranch tissues and their contribution to the hosts. In this study, the freshly laid eggs, which were almost completely dominated by a single unidentified genus of Sphingomonadaceae, exhibited a highly unique bacterial community distinct from that of other examined samples. Bacterium belonging to the Sphingomonadaceae was also found in the oviducal gland and dead eggs to a certain extent but was undetectable in seawater, suggesting the possibility of transgenerational microbial transfer from mother to embryo, as has been reported in little skate (Mika et al., 2021). The notably high occupancy of this bacterium in the freshly laid eggs raises the possibility of a symbiotic relationship with the host, although the absolute abundance of bacteria is extremely low. Several genera belonging to the family Sphingomonadaceae contain carotenoids that are known to act as antimicrobial agents (Kosako et al., 2015), and a carotenoid‐mediated antimicrobial mechanism may be active in the freshly laid eggs of catshark. Pseudomonas was observed in a considerable proportion of all catshark‐derived samples, although it was present at a very low proportion in freshly laid eggs. This genus also appears to be inherited from mother to offspring. Interestingly, Pseudomonas was reported to be consistently present at a high proportion of over 30% in the blood of the closely related small‐spotted catshark (*S. canicula*) (Muñoz‐Baquero et al., 2023) and was also detected at the highest proportion in the epidermal microbiome of the leopard shark (*Triakis semifasciata*) (Doane et al., 2023). These findings suggest that Pseudomonas may contribute to the metabolism of nitrogenous compounds, such as urea, which is abundant in marine cartilaginous fishes.

Based on the definition of ‘dead’ eggs in this study, we cannot rule out the possibility that these eggs were unfertilized. Thus, it is difficult to conclude that the proliferation of certain bacteria (e.g., Mycoplasma) observed in dead eggs had a harmful impact on embryonic survival. Most dead eggs, regardless of the time elapsed after oviposition, showed similar bacterial communities, with components shared with those in the oviducal gland epithelia. The explosive bacterial growth observed in several dead eggs may have been caused not by invasive pathogens from outside the capsule but likely by maternally derived bacteria utilizing the yolk diffused within the capsule as a nutrient source.

## CONCLUSIONS

This study provides the first direct evidence that early embryos of oviparous cloudy catshark are incubated in a clean intracapsular environment. This environment minimizes the number of microorganisms until pre‐hatching. The intracapsular environment of freshly laid eggs exhibited extremely low microbial diversity, largely dominated by an unidentified genus of Sphingomonadaceae. These findings enhance our understanding of embryonic protection mechanisms against pathogens. They may also offer a novel approach to exploring microbial associations with elasmobranch embryos.

## AUTHOR CONTRIBUTIONS

**Wataru Takagi:** Conceptualization; data curation; supervision; funding acquisition; investigation; writing – review and editing; writing – original draft; visualization; formal analysis; project administration. **Ayami Masuda:** Data curation; investigation; writing – review and editing. **Koya Shimoyama:** Writing – review and editing; resources; data curation. **Kotaro Tokunaga:** Writing – review and editing; resources. **Susumu Hyodo:** Writing – review and editing; supervision. **Yuki Sato‐Takabe:** Writing – review and editing; investigation; data curation; formal analysis; project administration.

## CONFLICT OF INTEREST STATEMENT

The authors declare no conflicts of interest.

## ETHICS STATEMENT

All animal experiments were approved by the Animal Ethics Committee of Atmosphere and Ocean Research Institute of the University of Tokyo (P19‐2). The present study was carried out in compliance with the ARRIVE guidelines.

## Supporting information


**Figure S1.** Alpha rarefaction curve of the samples. Red lines indicate the read count of the sample with the minimum number of reads (17,024 reads). The abbreviation is the same as those in Figure 4.
**Figure S2.** Significant associations of microbial taxa across different sample groups in comparison to freshly laid eggs identified by Maaslin2 (*q* < 0.05). The colour scale on the right indicates the direction and magnitude of the associations: red and blue represent higher and lower relative abundance to the freshly laid egg sample. The abbreviation is the same as those in Figure 4.

## Data Availability

The raw read data from 16S rRNA amplicon sequencing were deposited in the NCBI Sequence Read Archive (Accession No: DRR523913‐523937).
