# Supplementary material for: Low microbial abundance and community diversity in the egg capsule of the oviparous cloudy catshark (Scyliorhinus torazame) during oviposition
Source: Environ Microbiol Rep. 2024 Oct 22;16(5):e70025. doi: 10.1111/1758-2229.70025 (PMC11496042; doi:10.1111/1758-2229.70025)
Supplement: Supplementary file 1 — Figure S1. Alpha rarefaction curve of the samples. Red lines indicate the read count of the sample with the minimum number of reads (17,024 reads). The abbreviation is the same as those in Figure 4. Figure S2. Significant associations of microbial taxa across different sample groups in comparison to freshly laid eggs identified by Maaslin2 (q < 0.05). The colour scale on the right indicates the direction and magnitude of the associations: red and blue represent higher and lower relative abundance to the freshly laid egg sample. The abbreviation is the same as those in Figure 4. [file EMI4-16-e70025-s001.docx]

**Supplementary Figure 1**: Alpha rarefaction curve of the samples. Red lines indicate the read count of the sample with the minimum number of reads (17,024 reads). The abbreviation is the same as those in Figure 4.


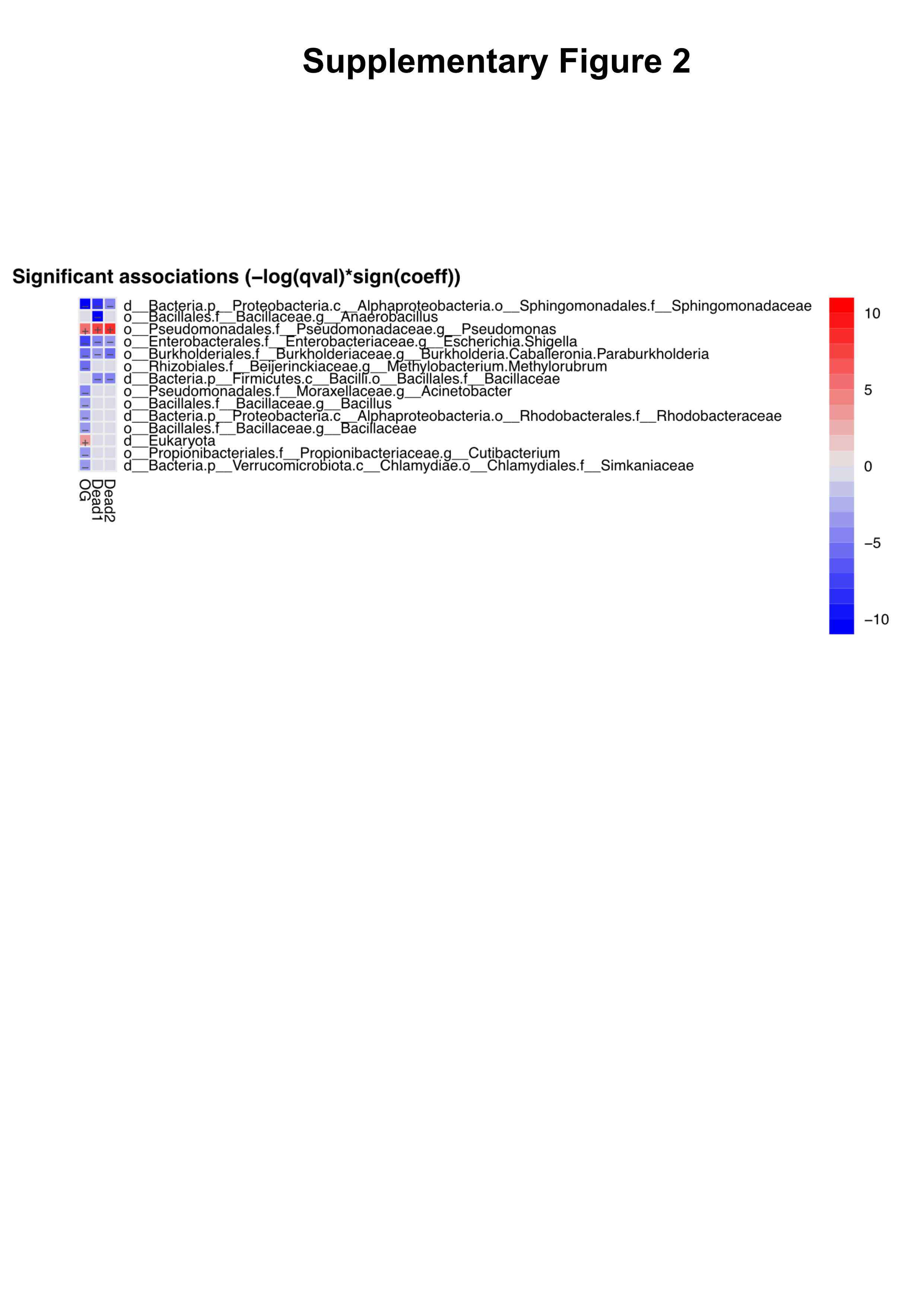


**Supplementary Figure 2**: Significant associations of microbial taxa across different sample groups in comparison to freshly laid eggs identified by Maaslin2 (*q* < 0.05). The color scale on the right indicates the direction and magnitude of the associations: red and blue represent higher and lower relative abundance to the freshly laid egg sample. The abbreviation is the same as those in Figure 4.
